# Fully automated segmentation in temporal bone CT with neural network: a preliminary assessment study

**DOI:** 10.1186/s12880-021-00698-x

**Published:** 2021-11-09

**Authors:** Jiang Wang, Yi Lv, Junchen Wang, Furong Ma, Yali Du, Xin Fan, Menglin Wang, Jia Ke

**Affiliations:** 1 Department of Otorhinolaryngology-Head and Neck Surgery, Peking University Third Hospital, Peking University, NO. 49 North Garden Road, Haidian District, Beijing, 100191 China; 2 School of Mechanical Engineering and Automation, Beihang University, Beijing, China

**Keywords:** Deep learning, Neural network, Automatic segmentation, Temporal bone CT, Accuracy

## Abstract

**Background:**

Segmentation of important structures in temporal bone CT is the basis of image-guided otologic surgery. Manual segmentation of temporal bone CT is time- consuming and laborious. We assessed the feasibility and generalization ability of a proposed deep learning model for automated segmentation of critical structures in temporal bone CT scans.

**Methods:**

Thirty-nine temporal bone CT volumes including 58 ears were divided into normal (n = 20) and abnormal groups (n = 38). Ossicular chain disruption (n = 10), facial nerve covering vestibular window (n = 10), and Mondini dysplasia (n = 18) were included in abnormal group. All facial nerves, auditory ossicles, and labyrinths of the normal group were manually segmented. For the abnormal group, aberrant structures were manually segmented. Temporal bone CT data were imported into the network in unmarked form. The Dice coefficient (DC) and average symmetric surface distance (ASSD) were used to evaluate the accuracy of automatic segmentation.

**Results:**

In the normal group, the mean values of DC and ASSD were respectively 0.703, and 0.250 mm for the facial nerve; 0.910, and 0.081 mm for the labyrinth; and 0.855, and 0.107 mm for the ossicles. In the abnormal group, the mean values of DC and ASSD were respectively 0.506, and 1.049 mm for the malformed facial nerve; 0.775, and 0.298 mm for the deformed labyrinth; and 0.698, and 1.385 mm for the aberrant ossicles.

**Conclusions:**

The proposed model has good generalization ability, which highlights the promise of this approach for otologist education, disease diagnosis, and preoperative planning for image-guided otology surgery.

## Introduction

The anatomy of the temporal bone is highly complex. It contains crucial structures including the facial nerve, cochlea, and ossicular chain, which are fine structures surrounded by a large amount of mastoid air cells [1]. Due to this complexity and large interpatient variation, it is often difficult for residents to recognize these structures at the 2-dimensional computed tomography (CT) level. Indeed, the anatomy of the temporal bone is one of the most challenging topics during the education of ENT surgeons [2]. In addition, prior to neurotologic surgery, clinicians require a broad and profound anatomical knowledge of the three- dimensional (3D) spatial relationships between risk structures and lesions to avoid complications, such as dizziness and facial nerve palsy [3, 4].

In recent years, novel technologies using image-guided neurotologic surgery have been developed such as minimally invasive cochlear surgery [5–8]. The tunnel from the mastoid cortex to the cochlea is drilled by a robot or micro-stereoscopic frame under imaging guidance [5–8]. Pre-surgical trajectory planning and intraoperative navigation require visualization of vital anatomic structures in 3D and extraction from pre-operative CT images, which require segmentation. Additionally, accurate image segmentation improves path planning safety and achieves the purpose of automated planning [8]. Manual segmentation is highly accurate; however, it is time-consuming and laborious method [6, 9]. Although some software tools that can enhance and accelerate the extraction of structures of interest have emerged, they still cannot achieve fully automatic segmentation [10]. Considering these aspects, it is necessary to automatically and accurately identify significant structures in temporal bone CT images.

The traditional automatic recognition of the anatomical structures of the temporal bone is mainly based on atlas-based segmentation [11–15]. The principle of this method is to use the volume that has been pre-segmented by doctors as an atlas to register other volume to be extracted, and appropriate structures are segmented by marking the transformed label map [14, 16]. However, important anatomical structures of the temporal bone such as ossicles, facial nerve, and labyrinth are ineffective for robust and accurate segmentation using pure atlas- based segmentation methods due to their small diameter, large interpatient variations, and/or the lack of local contrast [11, 12, 14]. At present, a compromise is achieved by combining refined strategies and atlas-based approaches to complete the automatic segmentation of the temporal bone [11, 12, 14, 17]. These versions of atlas-based segmentation are currently used for automatic segmentation of anatomical structures in the temporal bone and permit high segmentation accuracy in relatively normal anatomical structures. However, the performance of atlas-based methods is strongly related to the accuracy of registration, which is the primary limitation [13–15]. Further, large variations in patients’ anatomical structures, such as cochlear deformity, facial nerve displacement, and ossicle malformation preclude the identification of structures that match the traditional atlas in the area of interest. This will result in failure of automatic identification and segmentation.

Neural networks based on deep learning are widely used in medical imaging and pathological diagnosis, and have been reported to achieve satisfactory results in segmenting images [18–22]. Unlike traditional feature extraction, deep learning combines more complex architecture and internal feature extraction mechanisms to build a multilayer neural network, which may achieve higher accuracy and reproducibility in a fully automatic manner [23–25]. Furthermore, deep learning networks contain millions of mathematical function parameters, allowing machines to perform active learning according to the working mode of human neuronal networks [19, 20]. This implies that multilayered networks have large advantages for segmenting deformed anatomy. For this reason, deep learning and artificial neural networks have significantly improved medical image segmentation accuracy if given sufficient training data from the population [26–29]. However, few studies have focused on the segmentation of important structures using deep-learning networks in temporal bone CT images.

To address this gap in the field, we have proposed a deep-learning framework referred to as W-net architecture and performed preliminary training of the neural network to automatically segment critical structures in CT scans of normal adult conventional temporal bone [30]. To assess the feasibility of the model’s clinical application, we must further test the generalization ability of W-net. In this study, we used newly-collected temporal bone CT data and established two types of test sets, one representing normal structures and the other malformed. The performance of W-net was compared and analyzed with that of manual segmentation to evaluate the feasibility of the proposed model in clinical application.

## Materials and methods

### Data collection

Test Set data from 39 temporal bone CT volumes including 58 ears (including 20 ears with normal anatomy and 38 ears with deformity), were derived from routine scans of 39 subjects undergoing conventional temporal bone CT examinations (SIEMENS/ SOMATOM Definition Flash, German, 128-channel, thickness = 0.4 mm, pitch = 0.30 mm, pixel = 0.412 mm) in Peking University Third Hospital (Table 1). In this study, all CT volumes were newly-collected, thereby excluding the training model data. In the normal test set (n = 20), all CT data contained complete structures of the ossicle, facial nerve, and labyrinth. In the abnormal test set (n = 38), ossicular chain disruption (n = 10) and facial nerve covering the oval window (n = 10) were used to represent middle ear malformations, and Mondini dysplasia (n = 18) was used to represent inner ear (labyrinth) malformations. Patients underwent cross-sectional scans with base line connecting the infraorbital margin and external auditory meatus. The scanning range encompassed the region between the top border of the petrous bone and bottom boundary of the external auditory canal. Axial sections were obtained with the following settings: matrix size, 512 × 512; field of view, 220 × 220 mm; voltage, 120 kV; and current, 240 mA. Each registered patient underwent only one CT scan between February 2020 and May 2020. All CT images were downloaded from the physician’s workstation and saved in 512 × 512 pixel, DICOM format, for analysis. The voxel sizes of all CT data ranged from 0.4 * 0.4 * 0.4 mm^3^ to 0.5 * 0.5 * 0.4 mm^3^. An expert excluded cases of ear diseases, such as otitis media from temporal bone CT. This trial was approved by the Peking University Third Hospital Medical Ethics Committee.

**Table 1:** Numbers of ears, CT scans, and patients collected in study

	Ears	CT volumes	Patients
Normal groups	20	10	10
Ossicular chain disruption	10	10	10
Facial nerve covering the oval window	10	10	10
Mondini dysplasia	18	9	9
Total	58	39	39

### Construction of deep learning models

**Fig. 1:**
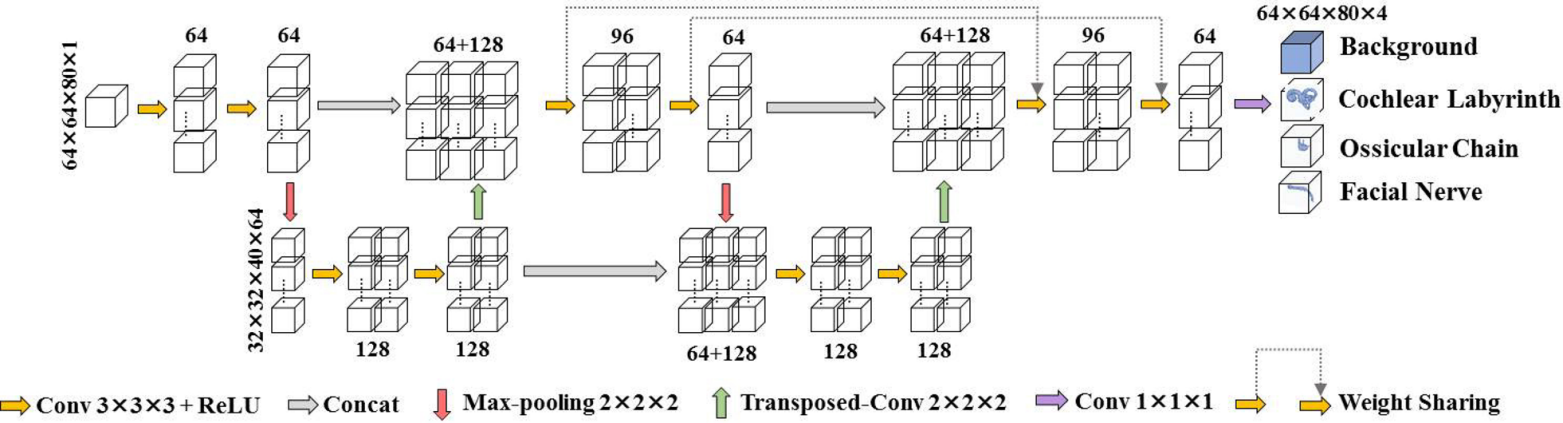
W-Net architecture. The number of channels is denoted next to the feature map

The proposed network model (W-Net) was described in detail in a previous study [30]. The W-net architecture in this study has no changes from the previously reported study [30]. Figure 1 shows the architecture of W-net. The deep learning network comprised two analysis paths and two synthesis paths, which performed the functions of analysis of temporal bone CT images and generation of full-resolution segmentation, respectively. The segmentation structures were located in the middle of CT imaging data, and the surrounding edges lacked segmentation target area. Thus, we set the pixel padding value in the 3D convolution operation of neural networks to 1, which ensured that the output and input dimensions of the network were the same (64 × 64 × 80 voxels). The number of channels for the first and second convolution of our deep-learning network was 1/2 and 1/3, respectively, to ensure a smooth transition. In this study, we selected the adaptive moment estimation (Adam) optimizer to optimize parameters via an iterative process [31]. A significant part of the network was the weighted cross entropy (WCE) as the loss function that could effectively solve the problems of small size and complex structure of the segmentation target and make the network fit faster.

### Experimental design

Our previous study used data enhancement technology and 30 temporal bone CT volumes (15 left, 15 right) to develop the neural network model, which allowed the architecture to locate and render important structures on the left or right sides of the temporal bone CT [30]. We have used 24 volumes (12 left, 12 right) and 6 volumes (3 left, 3 right) as the training and validation set respectively, and trained the W-net network through five fold cross-validation [30]. It should be noted that the test set in this study were not included the training and validation data set in the previous study [30].

In this study, we input the CT data of each group of normal temporal bone without labels into the neural network in DICOM format and allowed the framework to automatically segment and extract the facial nerve, ossicles, and labyrinth. Subsequently, we divided the CT data of abnormal structures into three portions, including ossicular chain disruption, facial nerve covering the oval window, and Mondini dysplasia, which were added to three folders in DICOM format. We imported the data in each folder separately into the neural network and automatically segmented the malformed structures. All experiments were performed on a workstation with a Xeon Silver 4110 CPU and a NVIDIA RTx2080Ti GPU (16G memory). Two cochlear implant surgeons manually delineated (consensus reviewed each individual CT volume), and reconstructed the relevant structures of interest in a slice-by-slice manner, and a medical image analysis scientist with over 30 years of clinical experience made final corrections for every structure. All segmentations were performed with the Mimics image processing software (Materialise NV, Leuven, Belgium, version 20.0). It should be noted that the annotators (two surgeons and one medical image analysis scientist) were blinded to the automated segmentation results.

### Evaluation

Using manual segmentation as the gold standard, we performed quantitative and qualitative evaluation of the results through automatic segmentation based on deep learning. In this study, Dice coefficient (DC) and Average symmetric surface distance (ASSD) were used as the evaluation criteria of segmentation accuracy to assess the congruence of automatic and manual segmentation [32–35]. The DC and ASSD were defined as follows:

$$DC(R,R_{0} ) = \frac{{2\left| {R \cap R_{0} } \right|}}{{\left| R \right| + \left| {R_{0} } \right|}}$$

$$\begin{aligned} & ASSD(R,R_{0} ) \\ & \quad = \frac{1}{{N_{R} + N_{{R_{0} }} }}\left\{ {\sum\limits_{{r \in R}} {\mathop {\min }\limits_{{r_{0} \in R_{0} }} (d(r,r_{0} ))} + \sum\limits_{{r_{0} \in R_{0} }} {\mathop {\min }\limits_{{r \in R}} (d(r,r_{0} ))} } \right\} \\ \end{aligned}$$

where *R* is the manually outlined mask developed by the clinicians, and *R*_*0*_ is the segmented mask using the deep learning method. *d* (*r,* *r*_*0*_) is the Euclidian distance between the two voxels. *r* and *r*_*0*_ are the surface points of *R* and *R*_*0*_, respectively. N_*R*_ and N_*R0*_ are the number of surface voxels on R and R_*0*_, respectively. The DC is spatial overlap index [36]. ASSD serves as a metric of shape similarity, which can supplement descriptions of structural margins [35]. Higher DC values (maximum value of 1) indicate that the automatic segmentation is more similar to ground truth. For ASSD, lower values (minimum value of 0) represent greater agreement between the automatic segmentation contours and gold standard.

## Results

### Neural network performance in the normal group

**Fig. 2:**
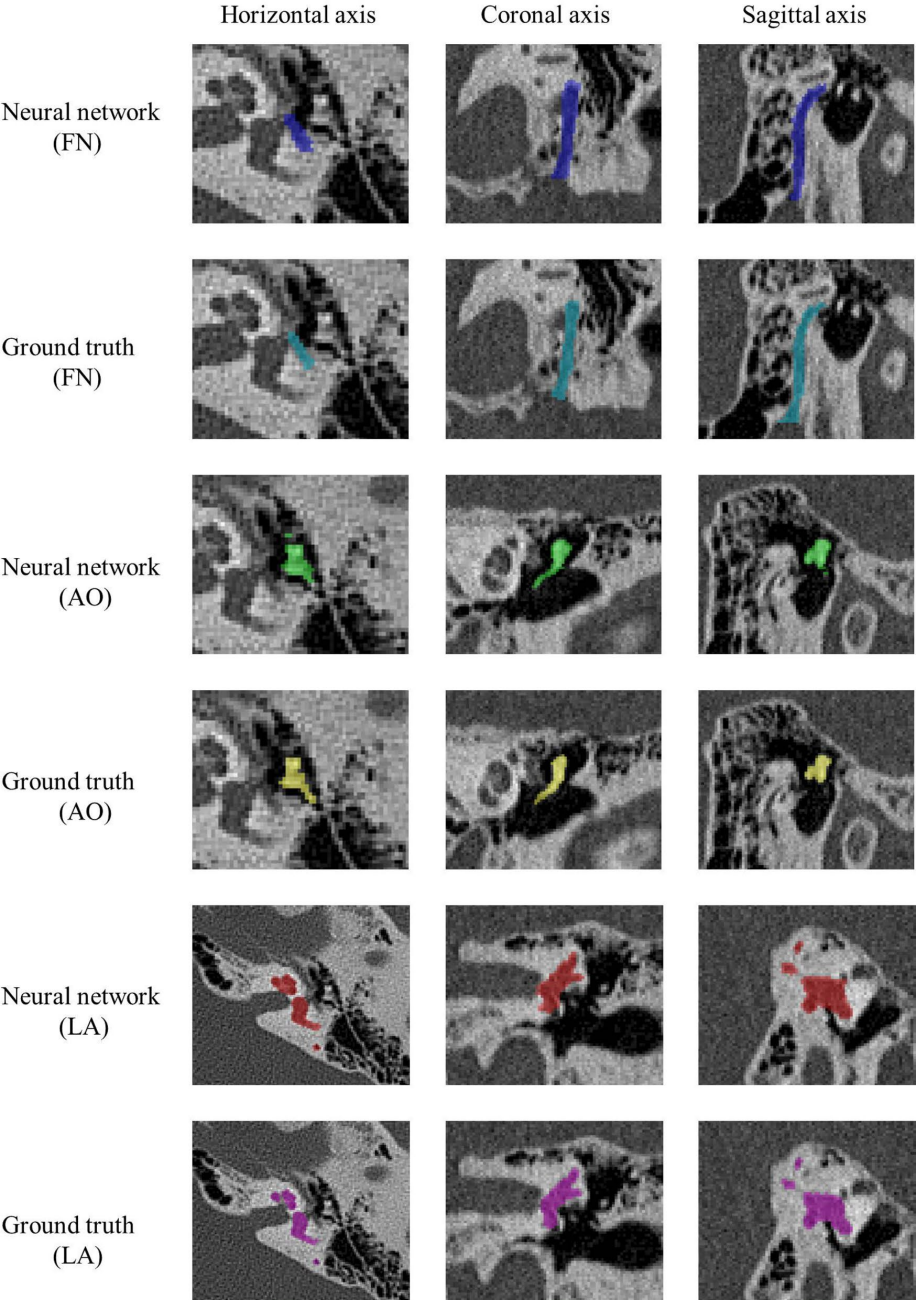
The masks of important structures generated by two methods in normal adult temporal bone CT images. Blue, green, and red labels denote the automatic extraction of FN, AO, and LA, respectively. Turquoise, yellow, and magenta labels represent the manual segmentation of FN, AO, and LA, respectively. CT, computed tomography; FN, facial nerve; AO, auditory ossicle; LA, labyrinth

**Fig. 3:**
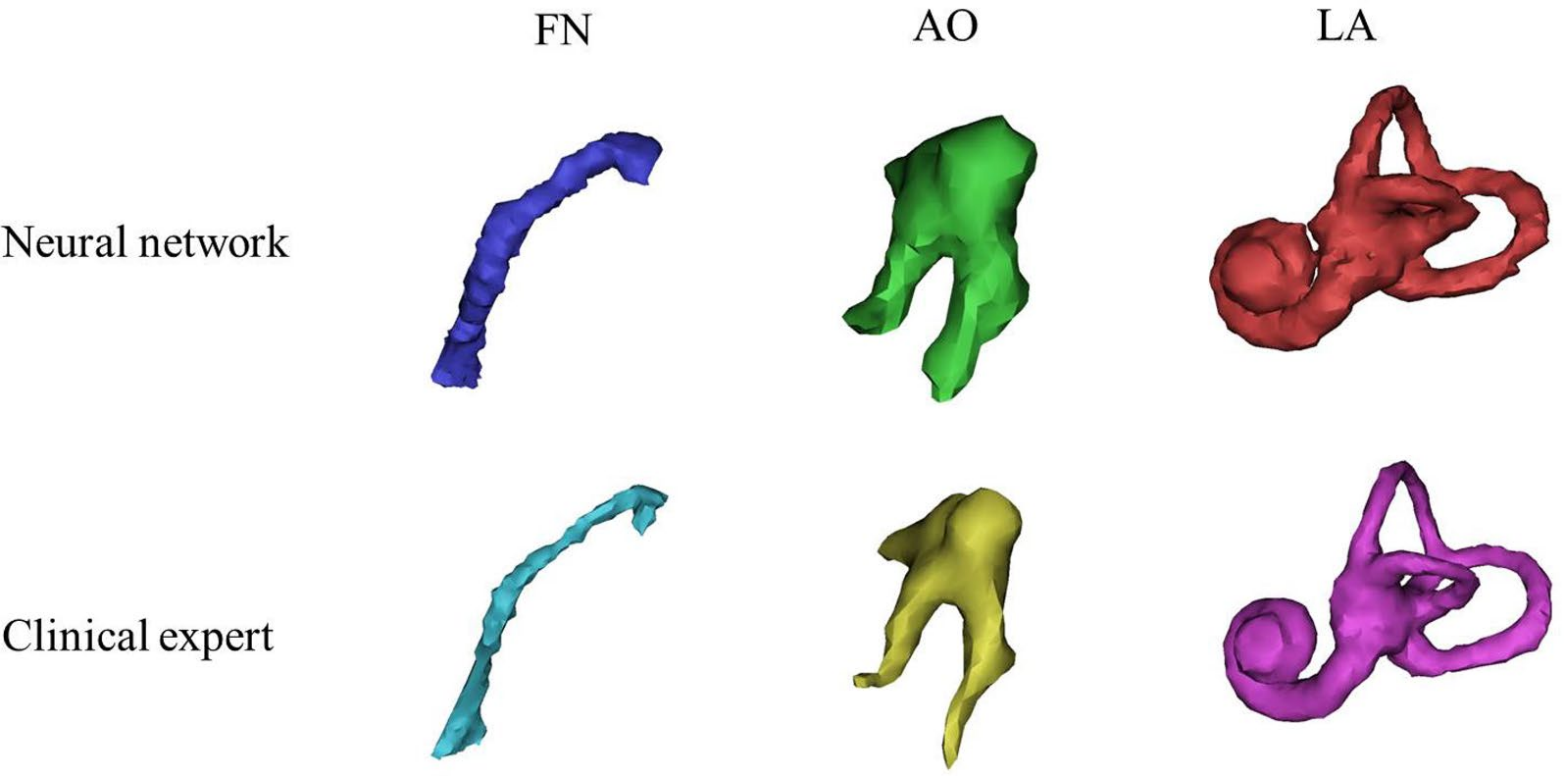
Three normal structures reconstructed by the neural network and clinical experts. FN, facial nerve; AO, auditory ossicle; LA, labyrinth

This method was tested on 20 normal ears. Figure 2 shows the masks of the facial nerve, ossicles, and labyrinth in normal adult CT images, which were manually annotated and automatically extracted. Figure 3 shows reconstructions of each structure including clinicians’ annotations and automatic segmentation. The labyrinth had the highest similarity, with slight differences in the vestibular and cochlear windows, which were connected to the middle ear cavity and lacked clear boundaries. The incudomalleolar joint was clearly visible, whereas the stapes was incomplete. The facial nerve exhibited the poorest performance and was merged with mastoid air cells in the vertical plane.

The results of automatic segmentation were compared with manually segmented images in a pixelwise manner. Table 2 summarizes the values of two metrics (DC and ASSD) for automatic segmentation of the normal adult dataset. For automatic segmentation of the labyrinth, DC and ASSD were 0.910 and 0.081 mm, respectively. The mean accuracy of ossicles was 0.855 for DC, and 0.107 mm for ASSD, respectively. For the facial nerve, deviations between automatic segmentation and the gold standard were noted, with DC and ASSD of 0.703 and 0.250 mm, respectively.

**Table 2:** The segmentation errors of the neural network for normal structures

	Dice index				Average symmetric surface distance (mm)			
	Max	Min	AVG	Mdn	Max	Min	AVG	Mdn
FN	0.838	0.592	0.703	0.687	0.355	0.114	0.250	0.260
AO	0.902	0.759	0.855	0.858	0.180	0.068	0.107	0.102
LA	0.926	0.880	0.910	0.910	0.162	0.054	0.081	0.069

FN, Facial Nerve; AO, Auditory Ossicle; LA, Labyrinth; AVG, Average; Mdn, Median

### Neural network performance in the abnormal group

**Fig. 4:**
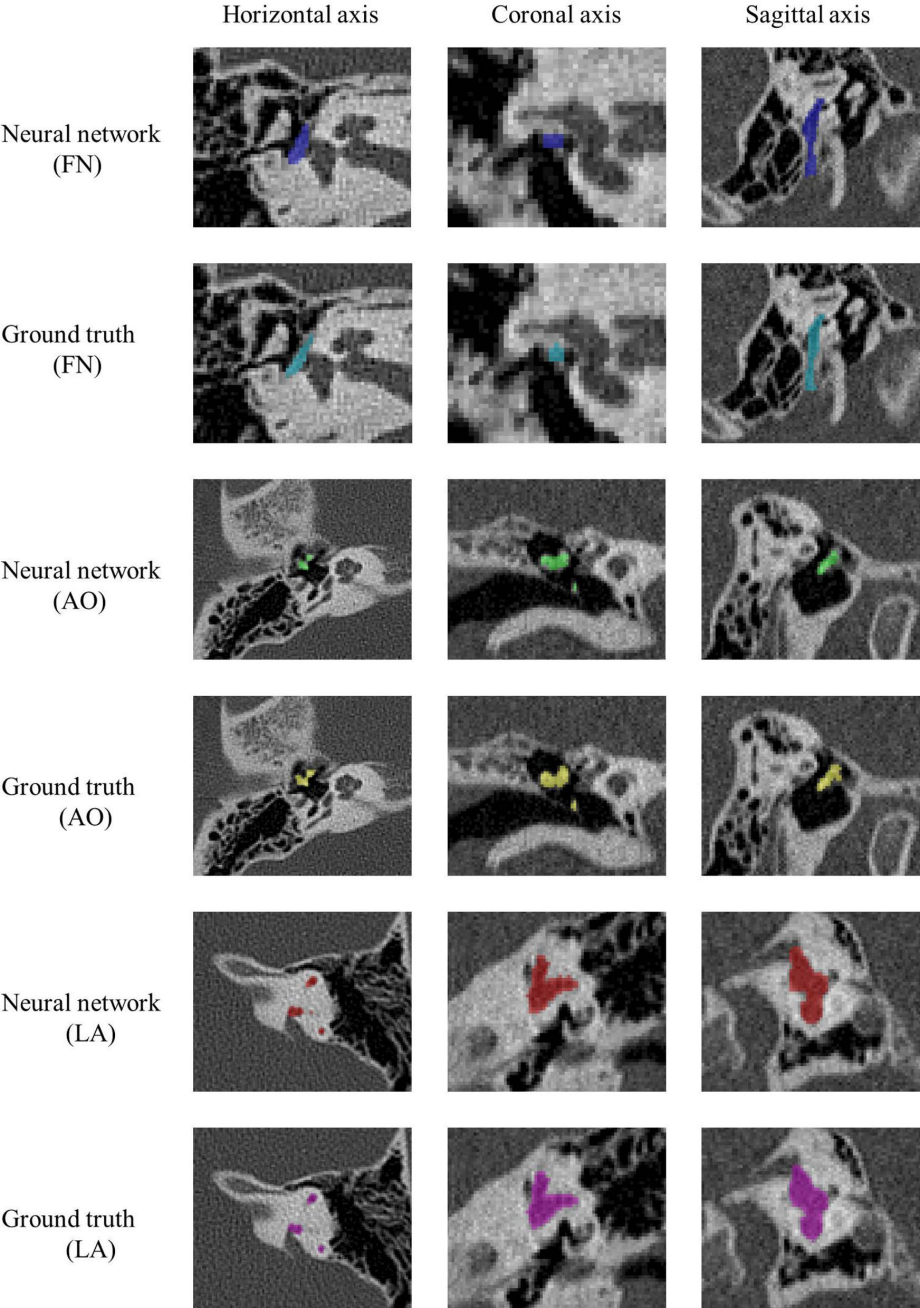
The masks of malformed structures created by two methods in abnormal temporal bone CT images. Blue, green, and red labels denote the FN (covering the vestibular window), AO (disruption), and LA (Mondini dysplasia), respectively. Turquoise, yellow, and magenta labels represent the manual segmentation of the FN (covering the vestibular window), AO (disruption), and LA (Mondini dysplasia), respectively. CT, computed tomography; FN, facial nerve; AO, auditory ossicle; LA, labyrinth

**Fig. 5:**
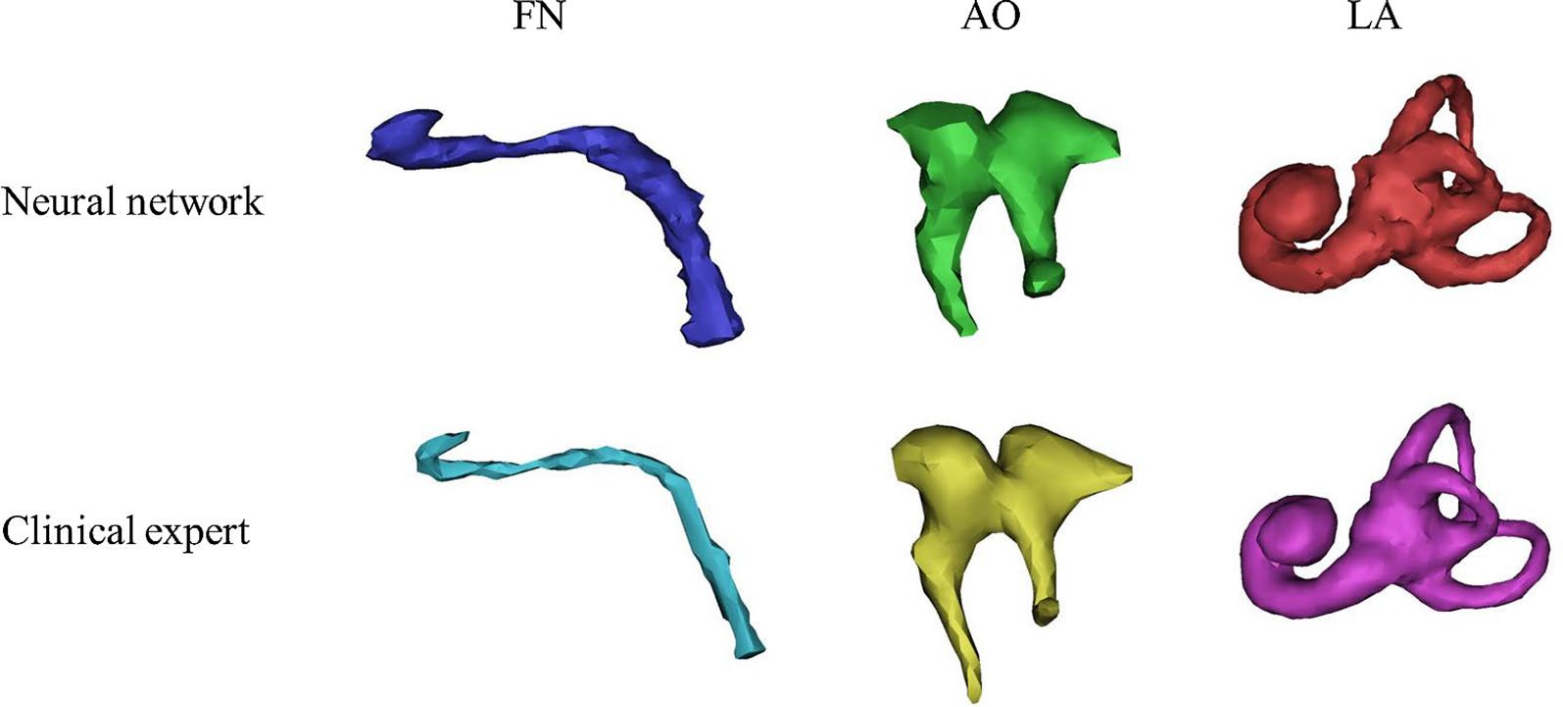
Three malformed structures reconstructed by the neural network and clinical experts. FN, facial nerve (covering the vestibular window); AO, auditory ossicle (disruption); LA, labyrinth (Mondini dysplasia)

Satisfactory results were obtained for malformed structures except facial nerve. Figure 4 shows the masks of malformed structures outlined by clinicians and the neural network in CT images. The 3D volume reconstructions of the structures segmented by the neural network and surgeons are shown in Fig. 5. Although the enlarged vestibular aqueduct was fused with the semicircular canals, the neural network clearly distinguished the margins and the labyrinth with high accuracy. For disrupted ossicular chains, the neural network accurately identified the malleus and incus, although some noise was present. However, the facial nerve covering the oval window was imperfectly annotated by the neural network.

The segmentation errors of the neural network based on different metrics are shown in Table 3. For dysplastic labyrinth, mean values for the labyrinth were 0.775 and 0.298 mm for DC and ASSD, respectively. The mean values of DC and ASSD for disrupted ossicular chains were 0.698 and 1.385 mm, respectively. For malformed facial nerves, the mean values of DC and ASSD were 0.506 and 1.049 mm, respectively.

**Table 3:** The segmentation errors of the neural network for malformed structures

	Dice index				Average symmetric surface distance (mm)			
	Max	Min	AVG	Mdn	Max	Min	AVG	Mdn
FN	0.638	0.373	0.506	0.511	2.174	0.332	1.049	0.817
AO	0.805	0.614	0.698	0.789	2.026	0.800	1.385	1.400
LA	0.894	0.345	0.775	0.838	0.673	0.144	0.298	0.247

FN, Facial Nerve; AO, Auditory Ossicle; LA, Labyrinth; AVG, Average; Mdn, Median

## Discussion

Neural network learning is prone to overfitting, that is, the model can correctly recognize the data in the training set but has poor performance of recognizing data outside the training set. Therefore, new data should be used for testing to evaluate the generalization ability of the neural network before clinical application. In this study, newly-collected temporal bone CT data were obtained to comprehensively evaluate the performance of automatic segmentation of normal and malformed structures using our proposed model.

The facial nerve has distinct characteristics including a small diameter, lack of local contrast, and large interpatient variation [11, 12].Indeed, even experienced otologists face difficulties in manual segmentation and determination of the exact boundaries of the facial nerve. During facial nerve segmentation, the geniculate ganglion and horizontal segment are easily confounded with the tensor tympani muscle, while the second genu and vertical segment are fused with mastoid air cells. Thus, it is not ideal to segment the facial nerve purely using atlas-based methods [13]. Noble and colleagues [11, 12, 37] combined an atlas-based method with a statistical model algorithm to segment the facial nerve in children and adult patients, whereby the mean errors of automatically and manually generated structures were 0.23 mm and 0.155 mm, respectively. Another report presented a novel atlas-based method that combined an intensity model and region-of-interest (ROI) masks, resulting in a DC value of 0.7 for the facial nerve [14]. Further, atlases of the facial nerve have been constructed on micro-CT to achieve highly accurate ground truth for test images, with a DC value over 0.75 [38, 39]. Recently, a new method based on deep learning was reported and the DC of facial nerve was as high as 0.8 from micro-CT [40]. However, in published research, ASSD has not yet been used to evaluate the segmentation accuracy of the facial nerve. In our study, the DC value of the normal facial nerve exceeded 0.7 and the ASSD value was 0.25 mm in conventional CT. The segmentation was fully automatic without involving surgeons. CT resolution may affect judgment and segmentation of the nerve margin. Additionally, the diameter of the facial nerve is only 3–5 voxels, and an error of 1–2 surface voxels may result in drastic changes in the DC value. Visual comparison of the results of automatic and manual segmentation revealed that most voxels overlapped perfectly, and the centerline of the facial nerve was consistent and reproducible.

The cochlea is an extremely sensitive structure in inner ear surgery, especially cochlear implant surgery. Previous research segmented the labyrinth to replace normal cochlear structure [6]. The cavity in the labyrinth has clear local contrast with the bone wall. However, the labyrinthine bone wall and temporal bone are completely fused together, and precise boundaries cannot be determined. As the cavity of labyrinth is surrounded by dense bone, the DC for the labyrinth was 0.8 based on a novel atlas-based method that united intensity model and ROI masks [14]. Li et al. reported that the DC values of the labyrinth (further subdivided into the cochlea, semicircular canal, and vestibule) were between 0.68 and 0.84, while the ASSD values were between 0.15 mm and 0.29 mm in conventional CT by deep learning network [35]. In this study, the normal and malformed labyrinth DC exceeded 0.90 and 0.77, respectively, while their ASSD were lower than 0.09 mm and 0.3 mm, respectively, indicating that our method was better than the atlas-based and other network methods in conventional CT. Generally, accurate segmentation of the intra-cochlear structures may help clinicians to precisely insert electrodes while reducing intra-cochlear damage during cochlear implantation [41]. However, the basilar and vestibular membranes are too thin for precise identification and location of intra-cochlear structures in conventional scans. To overcome this issue, Noble et al. [42] manually segmented the scala tympani and scala vestibuli to generate active shape models with higher resolution micro-CT, rendering the membranes visible. This model can be registered on the intra-cochlear regions of conventional CT and estimate the position of these cavities [43].The mean DC of the scala tympani and scala vestibuli was approximately 0.75 [42]. Indeed, errors in this method depend on the fidelity of the active shape model created, which is limited by the predefined deformation model. Given the encouraging performance of our W-net, employing our method to automatically segment the intra-cochlear structures in micro-CT and to accurately extract these cavities in conventional temporal bone CT will be the focus of our next study.

The ossicular chain consists of the malleus, incus, and stapes. In theory, ossicles are isolated in the middle ear cavity and surrounded by air, resulting in a very high local contrast, which results in the highest segmentation accuracy of the ossicular chain among all temporal bone structures. However, in our study, the DC values of both normal and malformed ossicles were lower than those for the labyrinth. Due to its small size, it was difficult to identify and describe the stapes in traditional temporal bone CT [1]. In the manual segmentation process, only a small portion of the stapes could be extracted, and it could not be delineated in some cases. The difficulty of delineating the stapes may have interfered with the performance of the neural network. Indeed, the DC of the stapes is significantly lower than that for other structures in the temporal bone [14]. Thus, the ossicular chain as a whole is often used for automatic segmentation evaluation [13, 44]. In this study, automatic segmentation via deep learning resulted in a DC greater than 0.85 for normal ossicles and an approximate value of 0.7 for deformed ossicles. This study's normal and malformed ossicles ASSD were lower than 0.11 mm and 1.4 mm, respectively. Compared with the results reported by Li et al., the accuracy of the ossicles segmented by our W-net method is slightly better than that of their neural network [35]. Recently, some groups employed more advanced synchrotron radiation phase-contrast imaging and micro-CT for temporal bone imaging, which demonstrated that the stapes could be clearly tracked and outlined [45]. With these novel radiological techniques, the ossicular chain can be more finely segmented.

Currently, there is no clear standard for the goal or allowance error of automatically segmenting temporal bone structure. All researchers hope that automatic segmentation can be closer to manual segmentation. In previous studies, we have tried to train W-net using CT datasets separately annotated by two surgeons [30]. If the metric values of the two models (trained from the two surgeons’ separately annotated CT dataset) are close, we consider that the accuracy of the automatic segmentation is close to the level of the surgeon. Therefore, we believe that the tolerable error of DC values for the facial nerve, ossicles, and labyrinth were 0.7, 0.8 and 0.85, respectively, in conventional temporal bone CT based on our experience. While the tolerable error of ASSD values for the facial nerve, ossicles, and labyrinth were 0.3 mm, 0.2 mm and 0.3 mm, respectively. It was noted that the automatic segmentation error should have different tolerant ranges according to various tasks such as image-guided cochlear access, mastoidectomy, and otologist education. Therefore, formulating detailed error standards for automatic segmentation of temporal bone based on various tasks will be one of our main goals in the future.

Structures delineated by automatic segmentation can be reconstructed by doctors via 3D visualization to assess positional relationships between vital structures and disease-affected regions, which serve as an important guide to surgeons for surgical planning. From teaching of surgical theories to medical practice, this approach will strengthen clinical education and enable students to better appreciate the 3D relationships of temporal bone anatomy [13]. Image-guided minimally invasive cochlear implant surgery requires segmentation of related structures and planning of safe paths before surgery. Automatically extracting these structures from CT image data will significantly reduce processing time and increase the convenience of path planning. Further, using this neural network to automatically segment the jugular fossa, carotid canal, internal acoustic meatus, sigmoid sulcus, and other structures is also under consideration. This automated segmentation approach can be used in other robot-assisted otological surgeries such as mastoidectomy and acoustic neuroma [46, 47].

This study has several limitations. First, in this study, we used a small sample size of normal and malformed structures to test the generalization ability of the proposed model, and provided a preliminary research basis for model parameter optimization and clinical application. In future experiments, a larger sample size of CT data with normal and abnormal structures will be used to further train and test the performance of the neural network. Second, the test set data in this study and the CT data in our previous study [30] were all from the same institution. In the future, we will collect CT volumes from various institutions, and different scales of CT images to further train and test our network model, which can improve the robustness and generalization ability of the W-net. Third, the single CT volumes annotated between different teams (observers) have a certain degree of variability, which may affect the performance of the neural network model during the training process. We will attempt to compare the differences between various annotators and observe the impact on W-net training. In addition, future training sets may include CT data from different annotators. Finally, with the exception of the CT data of the Mondini dysplasia from children, the data was primarily obtained from adult cases. In this regard, most congenital ear dysplasia and cochlear implant surgeries are performed in impuberal patients. The proposed approach should be applied to pediatric cases for further verification.

## Conclusions

In this study, we assessed the generalization ability of a novel method based on deep learning for automated segmentation of the facial nerve, labyrinth, and ossicles, which are relevant to otologic surgery. The accuracy of our method was close to manual segmentation of normal structures and abnormal labyrinth and ossicles, which indicates the promise of this approach for otologist education, disease diagnosis, and preoperative planning for image- guided otology surgery. In the future, we hope to apply this method for the segmentation of other temporal bone structures and skull base structures.

## Data Availability

The datasets analyzed during the current study are available from the corresponding author on reasonable request.

## Abbreviations

CT: Computed Tomography
DC: Dice coefficient
ASSD: Average symmetric surface distance
ENT: Ear, nose, and throat
3D: Three-dimensional
Adam: Adaptive moment estimation
WCE: Weighted cross entropy
ROI: Region-of-interest
FN: Facial nerve
AO: Auditory ossicle
LA: Labyrinth

## References

[1] Yamashita K, Hiwatashi A, Togao O, Kikuchi K, Matsumoto N, Momosaka D, Nakatake H, Sakai Y, Honda H (2018). Ultrahigh-resolution CT scan of the temporal bone. Eur Arch Otorhinolaryngol.

[2] Frithioff A, Sørensen MS, Andersen SAW (2018). European status on temporal bone training: a questionnaire study. Eur Arch Otorhinolaryngol.

[3] Isaacson B (2018). Anatomy and surgical approach of the ear and temporal bone. Head Neck Pathol.

[4] Bhutta MF (2016). A review of simulation platforms in surgery of the temporal bone. Clin Otolaryngol.

[5] Caversaccio M, Wimmer W, Anso J, Mantokoudis G, Gerber N, Rathgeb C, Schneider D, Hermann J, Wagner F, Scheidegger O, Huth M, Anschuetz L, Kompis M, Williamson T, Bell B, Gavaghan K, Weber S (2019). Robotic middle ear access for cochlear implantation: first in man. PLoS ONE.

[6] Ke J, Zhang SX, Hu L, Li CS, Zhu YF, Sun SL, Wang LF, Ma FR (2016). Minimally invasive cochlear implantation assisted by bi-planar device: an exploratory feasibility study in vitro. Chin Med J (Engl).

[7] Labadie RF, Noble JH (2018). Preliminary results with image-guided cochlear implant insertion techniques. Otol Neurotol.

[8] Bell B, Gerber N, Williamson T, Gavaghan K, Wimmer W, Caversaccio M, Weber S (2013). In vitro accuracy evaluation of image-guided robot system for direct cochlear access. Otol Neurotol.

[9] Wang J, Liu H, Ke J, Hu L, Zhang S, Yang B, Sun S, Guo N, Ma F (2020). Image-guided cochlear access by non-invasive registration: a cadaveric feasibility study. Sci Rep.

[10] Cardenas CE, Yang J, Anderson BM, Court LE, Brock KB (2019). Advances in auto-segmentation. Semin Radiat Oncol.

[11] Noble JH, Warren FM, Labadie RF, Dawant BM (2008). Automatic segmentation of the facial nerve and chorda tympani in CT images using spatially dependent feature values. Med Phys.

[12] Noble JH, Warren FM, Labadie RF, Dawant BM (2008). Automatic segmentation of the facial nerve and chorda tympani using image registration and statistical priors. Prog Biomed Opt Imaging – Proc SPIE.

[13] Noble JH, Dawant BM, Warren FM, Labadie RF (2009). Automatic identification and 3D rendering of temporal bone anatomy. Otol Neurotol.

[14] Powell KA, Liang T, Hittle B, Stredney D, Kerwin T, Wiet GJ (2017). Atlas-based segmentation of temporal bone anatomy. Int J Comput Assist Radiol Surg.

[15] Powell KA, Kashikar T, Hittle B, Stredney D, Kerwin T, Wiet GJ (2019). Atlas-based segmentation of temporal bone surface structures. Int J Comput Assist Radiol Sug.

[16] Kirsch V, Nejatbakhshesfahani F, Ahmadi SA, Dieterich M, Ertl-Wagner B (2019). A probabilistic atlas of the human inner ear's bony labyrinth enables reliable atlas-based segmentation of the total fluid space. J Neurol.

[17] Diao X, Chen S, Liang C, Yuanmei W (2005). 3D semi-automatic segmentation of the cochlea and inner ear. Conf Proc IEEE Eng Med Biol Soc.

[18] Carton FX, Chabanas M, Lann FL, Noble JH (2020). Automatic segmentation of brain tumor resections in intraoperative ultrasound images using U-Net. J Med Imaging (Bellingham).

[19] Wang YM, Li Y, Cheng YS, He ZY, Yang JM, Xu JH, Chi ZC, Chi FL, Ren DD (2020). Deep learning in automated region proposal diagnosis of chronic otitis media based on computed tomography. Ear Hear.

[20] Esteva A, Robicquet A, Ramsundar B, Kuleshov V, DePristo M, Chou K, Cui C, Corrado G, Thrun S, Dean J (2019). A guide to deep learning in healthcare. Nat Med.

[21] Ibtehaz N, Rahman MS (2020). MultiResUNet: rethinking the U-net architecture for multimodal biomedical image segmentation. Neural Netw.

[22] Kim YJ, Ganbold B, Kim KG (2020). Web-based spine segmentation using deep learning in computed tomography images. Healthc Inform Res.

[23] Kavur AE, Gezer NS, Barış M, Şahin Y, Özkan S, Baydar B, Yüksel U, Kılıkçıer Ç, Olut Ş, Bozdağı Akar G (2020). Comparison of semi-automatic and deep learning- based automatic methods for liver segmentation in living liver transplant donors. Diagn Interv Radiol.

[24] Wainberg M, Merico D, Delong A, Frey BJ (2018). Deep learning in biomedicine. Nat Biotechnol.

[25] Ding Y, Acosta R, Enguix V, Suffren S, Ortmann J, Luck D, Dolz J, Lodygensky GA (2020). Using deep convolutional neural networks for neonatal brain image segmentation. Front Neurosci.

[26] Chen C, Qin C, Qiu H, Tarroni G, Duan J, Bai W, Rueckert D (2020). Deep learning for cardiac image segmentation: a review. Front Cardiovasc Med.

[27] Caballo M, Pangallo DR, Mann RM, Sechopoulos I (2020). Deep learning-based segmentation of breast masses in dedicated breast CT imaging: radiomic feature stability between radiologists and artificial intelligence. Comput Biol Med.

[28] Winkel DJ, Weikert TJ, Breit HC, Chabin G, Gibson E, Heye TJ, Comaniciu D, Boll DT (2020). Validation of a fully automated liver segmentation algorithm using multi-scale deep reinforcement learning and comparison versus manual segmentation. Eur J Radiol.

[29] Rad RM, Saeedi P, Au J, Havelock J (2020). Tropjhrctoderm segmentation in in human embryo images via inceptioned U-Net. Med Image Anal.

[30] Lv Y, Ke J, Xu Y, Shen Y, Wang J, Wang J (2021). Automatic segmentation of temporal bone structures from clinical conventional CT using a CNN approach. Int J Med Robot.

[31] Kingma DP, Ba J. Adam: a method for stochastic optimization. Computer Science. 2014. https://arxiv.org/pdf/1412.6980.

[32] Rashed EA, Gomez-Tames J, Hirata A (2020). End-to-end semantic segmentation of personalized deep brain structures for non-invasive brain stimulation. Neural Netw.

[33] Dice LR (1945). Measures of the amount of ecologic association between species. Ecology.

[34] Park J, Yun J, Kim N, Park B, Cho Y, Park HJ, Song M, Lee M, Seo JB (2020). Fully automated lung lobe segmentation in volumetric chest CT with 3D U-Net: validation with intra- and extra-datasets. J Digit Imaging.

[35] Li X, Gong Z, Yin H, Zhang H, Wang Z, Zhuo L (2020). A 3D deep supervised densely network for small organs of human temporal bone segmentation in CT images. Neural Netw.

[36] Polanski WH, Zolal A, Sitoci-Ficici KH, Hiepe P, Schackert G, Sobottka SB (2020). Comparison of automatic segmentation algorithms for the subthalamic nucleus. Stereotact Funct Neurosurg.

[37] Reda FA, Noble JH, Rivas A, McRackan TR, Labadie RF, Dawant BM (2011). Automatic segmentation of the facial nerve and chorda tympani in pediatric CT scans. Med Phys.

[38] Gare BM, Hudson T, Rohani SA, Allen DG, Agrawal SK, Ladak HM (2020). Multi-atlas segmentation of the facial nerve from clinical CT for virtual reality simulators. Int J Comput Assist Radiol Sug.

[39] Lu P, Barazzetti L, Chandran V, Gavaghan K, Weber S, Gerber N, Reyes M (2018). Highly accurate facial nerve segmentation refinement from CBCT/CT imaging using a super-resolution classification approach. IEEE Trans Biomed Eng.

[40] Nikan S, Agrawal SK, Ladak HM. Fully automated segmentation of the temporal bone from micro-CT using deep learning. In: Proceedings of SPIE 11317, medical imaging 2020: biomedical applications in molecular, structural, and functional imaging, 113171U (28 February 2020). 10.1117/12.2549609.

[41] Skinner MW, Holden TA, Whiting BR, Voie AH, Brunsden B, Neely JG, Saxon EA, Hullar TE, Finley CC (2007). In vivo estimates of the position of advances bionics electrode arrays in the human cochlea. Ann Otol Rhinol Laryngol Suppl.

[42] Noble JH, Labadie RF, Majdani O, Dawant BM (2011). Automatic segmentation of intracochlear anatomy in conventional CT. IEEE Trans Biomed Eng.

[43] Noble JH, Labadie RF, Gifford RH, Dawant BM (2013). Image-guidance enables new methods for customizing cochlear implant stimulation strategies. IEEE Trans Neural Syst Rehabil Eng.

[44] Fauser J, Stenin I, Bauer M, Hsu WH, Kristin J, Klenzner T, Schipper J, Mukhopadhyay A (2019). Toward an automatic preoperative pipeline for image-guided temporal bone surgery. Int J Comput Assist Radiol Surg.

[45] Elfarnawany M, Rohani SA, Ghomashchi S, Allen DG, Zhu N, Agrawal SK, Ladak HM (2017). Improved middle-ear soft-tissue visualization using synchrotron radiation phase-contrast imaging. Hear Res.

[46] McBrayer KL, Wanna GB, Dawant BM, Balachandran R, Labadie RF, Noble JH (2017). Resection planning for robotic acoustic neuroma surgery. J Med Imaging (Bellingham).

[47] Dillon NP, Balachandran R, Siebold MA, Webster RJ, Wanna GB, Labadie RF (2017). Cadaveric testing of robot-assisted access to the internal auditory canal for vestibular schwannoma removal. Otol Neurotol.

